# Combined unsupervised and semi-automated supervised analysis of flow cytometry data reveals cellular fingerprint associated with newly diagnosed pediatric type 1 diabetes

**DOI:** 10.3389/fimmu.2022.1026416

**Published:** 2022-10-27

**Authors:** Camillo Bechi Genzano, Eugenia Bezzecchi, Debora Carnovale, Alessandra Mandelli, Elisa Morotti, Valeria Castorani, Valeria Favalli, Angela Stabilini, Vittoria Insalaco, Francesca Ragogna, Valentina Codazzi, Giulia Maria Scotti, Stefania Del Rosso, Benedetta Allegra Mazzi, Maurizio De Pellegrin, Andrea Giustina, Lorenzo Piemonti, Emanuele Bosi, Manuela Battaglia, Marco J. Morelli, Riccardo Bonfanti, Alessandra Petrelli

**Affiliations:** ^1^ Diabetes Research Institute, IRCCS Ospedale San Raffaele, Milan, Italy; ^2^ Center for Omics Sciences, IRCCS Ospedale San Raffaele, Milan, Italy; ^3^ Department of Pediatrics, IRCCS Ospedale San Raffaele, Milan, Italy; ^4^ Department of General Medicine, Diabetes and Endocrinology, IRCCS Ospedale San Raffaele, Milan, Italy; ^5^ Laboratory Medicine, Autoimmunity Section, IRCCS Ospedale San Raffaele, Milan, Italy; ^6^ Immuno-Hematology and Transfusion Medicine (ITMS), IRCCS Ospedale San Raffaele, Milan, Italy; ^7^ Pediatric Orthopedic and Traumatology Unit, IRCCS Ospedale San Raffaele, Milan, Italy; ^8^ Institute of Endocrine and Metabolic Sciences, IRCCS Ospedale San Raffaele, Milan, Italy; ^9^ Università Vita-Salute San Raffaele, Milan, Italy

**Keywords:** type 1 diabetes, fingerprints, immune markers, pediatric diabetes, flow cytometry, computational biology

## Abstract

An unbiased and replicable profiling of type 1 diabetes (T1D)-specific circulating immunome at disease onset has yet to be identified due to experimental and patient selection limitations. Multicolor flow cytometry was performed on whole blood from a pediatric cohort of 107 patients with new-onset T1D, 85 relatives of T1D patients with 0-1 islet autoantibodies (pre-T1D_LR), 58 patients with celiac disease or autoimmune thyroiditis (CD_THY) and 76 healthy controls (HC). Unsupervised clustering of flow cytometry data, validated by a semi-automated gating strategy, confirmed previous findings showing selective increase of naïve CD4 T cells and plasmacytoid DCs, and revealed a decrease in CD56^bright^NK cells in T1D. Furthermore, a non-selective decrease of CD3^+^CD56^+^ regulatory T cells was observed in T1D. The frequency of naïve CD4 T cells at disease onset was associated with partial remission, while it was found unaltered in the pre-symptomatic stages of the disease. Thanks to a broad cohort of pediatric individuals and the implementation of unbiased approaches for the analysis of flow cytometry data, here we determined the circulating immune fingerprint of newly diagnosed pediatric T1D and provide a reference dataset to be exploited for validation or discovery purposes to unravel the pathogenesis of T1D.

## Introduction

Type 1 diabetes is a chronic autoimmune disease where the interaction between genes and environment shapes an immune-mediated attack of pancreatic β cells, eventually leading to chronic hyperglycemia and lifelong insulin dependence (1–3). Islet autoantibodies (Aabs) (4) mark the development of autoimmunity, so that the presence of ≥2 Aab in relatives of patients with type 1 diabetes is a highly sensitive predictor of the development of symptomatic diabetes (5), with a risk rising to almost 100% in pediatric subjects (6). However, while islet Aabs appear to be innocent bystanders in the development of type 1 diabetes, the chronic damage of insulin-producing β cells has historically been attributed to autoreactive T cells (7).

The development of type 1 diabetes has been associated with altered frequency and phenotype of both innate and adaptive immune cells (8), with a prominent role ascribed to subpopulations such as monocytes, dendritic cells (DCs), natural killer cells (NK cells), B cells and T cells, including regulatory T cells (Tregs) (9–15). However, despite numerous reports showing alterations of the circulating immune profile in patients with newly diagnosed type 1 diabetes, the technical limitations of cell analysis techniques may have prevented the identification of clinically relevant immune cell subsets with a “pro-diabetogenic” potential. Indeed, analysis of data obtained with multiparameter flow cytometry can be complex, as they are operator-dependent, ultimately affecting the quality and type of results. Furthermore, previous studies often lack proper control populations, preferring age and sex-matched healthy donors rather than relatives of patients with type 1 diabetes without persistent autoimmunity—but with a common genetic background— or patients with other autoimmune diseases. All these methodological biases have contributed to the generation of contradictory results and lack of data replicability.

In the present study, by validating unsupervised analysis of flow cytometry data with a semi-automated gating strategy and including proper control populations, we identify the peripheral cellular fingerprint of pediatric patients with newly diagnosed type 1 diabetes. Furthermore, we show that the frequency of naïve (N) CD4 T cells at disease onset is associated with partial remission, thus playing a potential role in a later stage of the autoimmune response.

## Materials and methods

### Subjects and data collection

A cohort of 326 five to eighteen-year-old children was enrolled at IRCCS Ospedale San Raffaele (OSR) from September 2018 to June 2021. The subject population was distributed as follows: 107 patients with newly diagnosed type 1 diabetes (T1D); 85 relatives of patients with type 1 diabetes (pre-T1D_LR) defined at low-risk of developing the disease as 0 or 1 islet Aab were detected; 58 patients with celiac disease or autoimmune thyroiditis (CD_THY) and 76 healthy controls (HC). The study was approved by the San Raffaele Hospital Ethics Committee (protocol TIGET004-DRI003). **Table 1** describes the demographics and clinical characteristics of the four groups of individuals enrolled in this study. Exclusion criteria for the participation to the study were: age <5 and >18 years, antibiotic therapy in progress or concluded in the last 10 days, signs of infection (fever, cough, rhinitis) in the last 2 weeks. Children diagnosed with type 1 diabetes were hospitalized in the Pediatric Department of OSR; blood samples were collected between 5 and 10 days after diagnosis. First- and second degree relatives of patients with type 1 diabetes with <2 detectable Aabs, which confers a low risk of developing the disease (16), were enrolled in this study. These individuals were enrolled in the Type 1 Diabetes TrialNet Pathway to Prevention Trial (TN01) (17) at the TrialNet Clinical Center of the San Raffaele Hospital. The complete TN01 protocol is available online (18). The study was approved by the OSR Ethics Committee (IRB# NHPROT32803-TN01). Seventy-six healthy non-diabetic children with no family history for type 1 diabetes were recruited at the pediatric Day hospital and Day surgery facilities of OSR, where they were admitted for suspected growth disorders or to undergo surgery for congenital diseases (i.e., flatfeet or hallux valgus). Fifty-eight children previously diagnosed with celiac disease (n=29) or autoimmune thyroiditis (n=29) who had a follow-up visit at the pediatric outpatient clinic of OSR were enrolled in this study. Detection of the islet Aabs GADA, IA2, ICA, mIAA, and ZnT8 was performed on subjects from all groups. The partial clinical remission of type 1 diabetes was assessed in 58 patients 12.2 months (IQR 11.1-13.2) after disease onset, using the insulin dose adjusted HbA1c (IDAA1c) calculated as HbA1c (%) + 4×insulin dose (U/kg per 24 hours); an IDAA1c equal to or less than 9 indicates the partial remission period (19). For the longitudinal analysis of relatives of patients with type 1 diabetes with pre-symptomatic stages of the disease, 2 groups of individuals enrolled in the TN01 trial who had cryopreserved PBMCs available in the local biobank were selected: 8 individuals who were Stage 0 (as they had 0 or 1 Aab) at T0 observation and persisted in Stage 0 at T1 observation; and 7 individuals that from Stage 0 at T0 transitioned to Stage ≥1 at T1 – i.e., developed two or more Aabs. Individuals from the two groups were sex- and age-matched (data not shown).

**Table 1:** Characteristics of the study cohort.

	HC N=76	CD_THY N=58 (29 CD, 29 THY)	preT1D_LR N=85	T1D N=107
Age (years) (median - IQR)	13.8 (12.7 -14.9)	12.6 (9.8 -14.8)	12.9 (10.8 -15.5)	11.0 (9.0 -13.4)
Gender (M/F)	41/35 M=54%	16/42 M=28%	43/42 M=51%	66/41 M=62%
BMIp (median - IQR)	64.9 (22.9-77.83)	41.6 (23.7-68.3)	72.4 (30.95-82.72)	42.0 (6.4-73.1)
HbA1c (mmol/L) (median - IQR)	NA	NA	NA	103.0 (87.0-117.0)
n. of islet Aabs	0 Aab=74/76 1 Aab=2/76	0 Aab=50/53 1 Aab=2/53 2 Aab=1/53	0 Aab=77/85 1 Aab=8/85	0 Aab=3/107 1 Aab=16/107 2 Aab=32/107 >3 Aab=56/107
Anti-TPO	NA	10/11 THY 1/22 CD	NA	16/107
Anti-TG	0/59	1/7 THY 26/29 CD	2/84	15/107
HLA_DR3 and/or DR4	9/54	27/35	48/85	73/85
WBC (10x3 ul) (median - IQR)	6.1 (5.1-7.5)	6.0 (5.2-7.1)	6.5 (5.7-8.0)	5.7 (4.8-6.6)
Neutrophils (median - IQR)	3.0 (2.4-4.0)	3.1 (2.4-3.6)	3.3 (2.6-4.2)	2.3 (1.8-2.8)
Monocytes (median - IQR)	0.5 (0.4-0.6)	0.5 (0.4-0.6)	0.5 (0.5-0.62)	0.50 (0.40-0.59)
Lymphocytes (median - IQR)	2.2 (1.9-2.7)	2.2 (1.7-2.8)	2.5 (2.0-3.0)	2.6 (2.10-3.05)

Clinical and laboratory characteristics of healthy controls (HC), subjects with celiac or thyroid diseases (CD_THY), relatives of T1D patients with 0-1 autoantibodies (preT1D_LR), and subjects with type 1 diabetes (T1D). WBC, Neutrophils, Lymphocytes, Monocytes are expressed as 10x3/ul. BMIp, Body Mass Index for age percentile; Aabs, Autoantibodies; TPO, Thyroid peroxidase; TG, Transglutaminase; WBC, White blood cells; NA, not available.

### Preparation of cells for flow cytometry

Whole blood was collected into a Vacuette^®^ blood collection tube with ACD-B anticoagulant solution (Greiner). After red blood cell lysis, the sample was washed and stained with a mix of monoclonal antibodies based on the specific panel. The list of anti-human monoclonal antibodies, including information on the company catalogue number, clone and concentration used for our assays is provided in **Supplementary Table 1**. Five panels of antibodies were designed and labelled as T cells, T&NK cells, B cells, Tregs and DCs/monos (**Supplementary Figure 1A**) encompassing main subsets of T cells, NK cells, B cells, Tregs, DCs and monocytes detected using 26 surface markers and the intracellular marker forkhead box P3 (FoxP3); for the Treg panel, intracellular staining was performed after fixation and permeabilization. The five panels were performed on all the blood samples; however, some samples are missing individual values due to technical errors, as indicated in **Supplementary Figure 1B**. In the longitudinal analysis, N CD4 T cells were identified using the following markers: CD3, CD4, CD45RA and CCR7. Cells were acquired on a BD FACSCanto-II flow cytometer equipped with FACSDiva software (Becton Dickinson, Franklin Lakes, NJ) within 24 hours. Rainbow calibration particles (Spherotech Inc., Lake Forest, IL) were used to calibrate and normalize acquisition settings in each experiment.

### Unsupervised clustering and semi-automated supervised gating

Unsupervised analysis and visual representation of cell populations was realized using the FlowSOM algorithm (20), included in the CyTOF/CATALYST pipeline (version 1.14.1) (21). Clusters showing similar marker expression profiles were manually merged. Collectively we identified 52 cell clusters, including 5 unclassified and 1 duplicate cluster (**Supplementary Figure 2 A-E**). Next, for data visualization, we applied the non-linear dimensionality reduction technique UMAP to the lineage marker levels, selecting a maximum of 500 cells per sample, using the *runDR()* function included in CyTOF workflow (22). To validate unsupervised clustering results, and to minimize manual biases, we set up a semi-automated supervised gating strategy using the OpenCyto R package (23) (gating strategies are shown in **Supplementary Figures 3-4**). Methodological details regarding unsupervised clustering and semi-automated supervised gating are contained in the **Supplemental Material**.

### Statistics

Cell populations were compared between the four groups using a linear model by adding age, sex and operator variability as covariates. P-values were determined by performing the multiple pairwise-comparisons with Tukey *post-hoc* test, using the function glht() in multcomp R package (version 1.4-19). The correlation between the frequencies of cell populations identified through unsupervised and semi-automated supervised analysis was performed using the Spearman’s rank-order correlation. Principal component analysis (PCA) (24) was performed on the circulating immunome, according to the frequencies of the 46 immunological populations identified by the unsupervised analysis (excluding unclassified and duplicate clusters). In all presented boxplots, medians are shown. The “hinges” represent the first and third quartile. The “whiskers” are the smallest and largest values without considering the outliers. To assess the frequency of CD3^+^CD56^+^ regulatory T cells, P-values were calculated by performing Students T test and Tukey *post-hoc* test for pairwise and multiple comparison respectively. A receiver operating characteristic (ROC) curve based on binary logistic regression was performed on 58 T1D subject to assess the predictive potential of the baseline frequency of N CD4 T cells on partial remission. In the longitudinal analysis, the Wilcoxon matched-pairs signed ranks test was used to analyze differences between paired samples. Statistical analyses were performed in R environment (version 4.0.3) and plotting was done using the ggplot2 R package (version 3.3.6). P-values less than 0.05 were regarded as statistically significant.

## Results

### Unsupervised clustering and semi-automated supervised analysis show an increase of N CD4 T cells and pDCs and a decrease of CD56^bright^ NK cells in newly diagnosed type 1 diabetes

**Figure 1:**
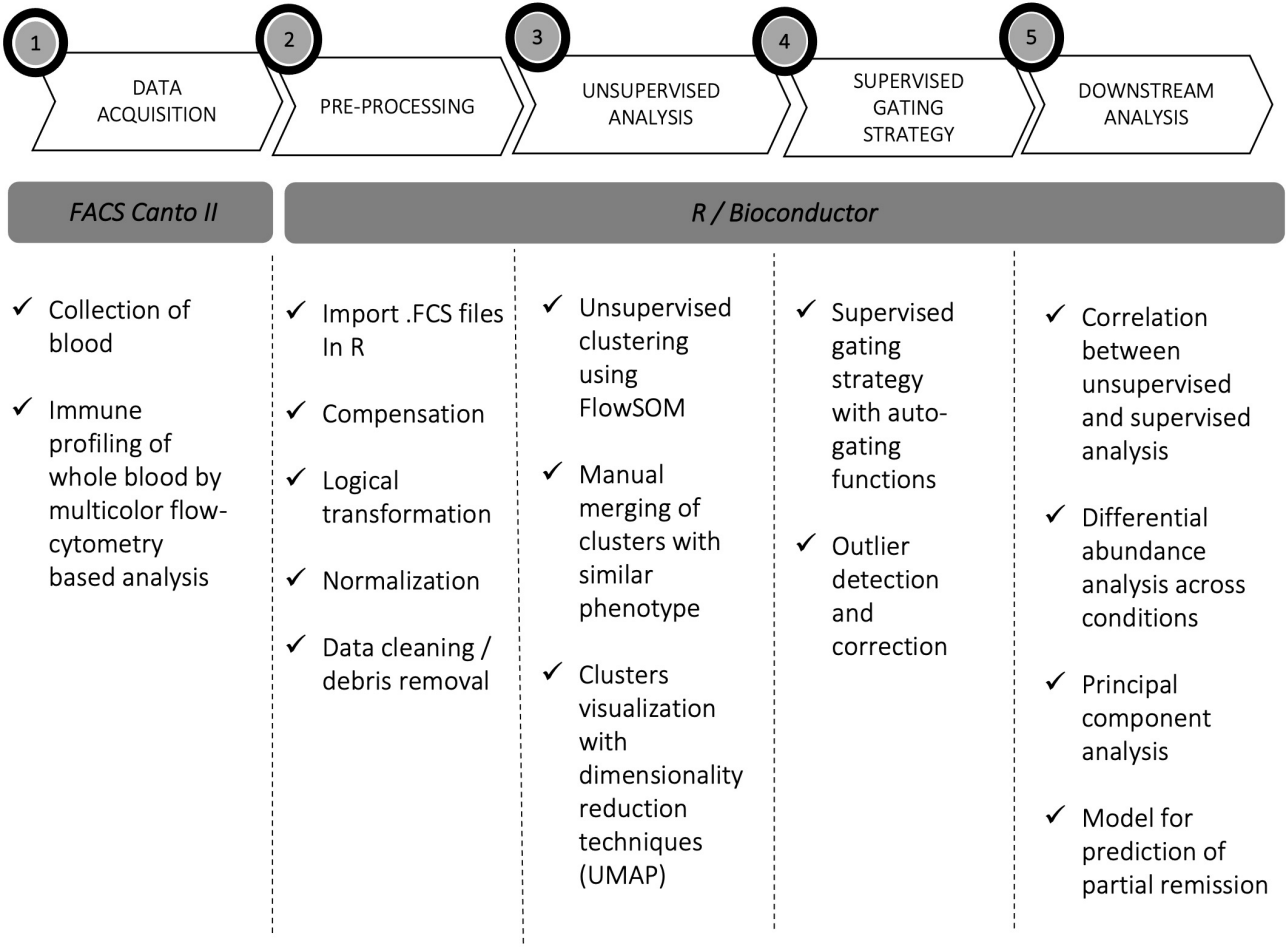
Experimental workflow. Experimental workflow of the study: acquisition (1), pre-processing (2), processing (3, 4), and downstream data analysis (5).

Unsupervised and semi-automated supervised analyses of five flow cytometry panels designed for the characterization of T cells, B cells, NK cells, DCs and monocytes were performed on whole blood of pediatric newly diagnosed T1D patients and on HC, CD_THY and pre-T1D_LR as control populations. **Figure 1** illustrates the experimental workflow of the study. Unsupervised analysis allowed the identification of 46 immune cell clusters, whose frequency within HC is shown in **Table 2**. A very high overall correlation (r = 0.94) was found between unsupervised computational clustering and semi-automated analysis (**Supplementary Figure 5A**), with only 5 cell populations – i.e., CD45RA^-^ FoxP3^lo^ non Tregs, CM CD4 T cells, CD45RA^+^ FoxP3^lo^ resting Tregs, unswitched memory B cells and non-classical monocytes – displaying a correlation coefficient < 0.5 (**Supplementary Figure 5B**).

**Table 2:** Cell frequencies in healthy children.

Parent	Population	1st Qu.	Median	3rd Qu.	Mean
Lymphocytes	T cells	71.65%	75.67%	79.11%	74.99%
	B cells	10.93%	13.42%	17.12%	13.82%
Lympho-monocytes	HLADR+LIN-	10.55%	13.39%	16.15%	14.11%
T cells	CD4 T	54.21%	59.73%	65.17%	59.47%
	CD8 T	24.27%	28.46%	32.03%	28.49%
	CD4+ CD8+ T	0.26%	0.38%	0.54%	0.50%
	CD4- CD8- T	8.55%	10.68%	14.27%	11.73%
	CD3+ CD56+ T	1.93%	3.10%	5.02%	4.19%
CD4 T cells	CM CD4 T	9.43%	11.83%	13.69%	11.85%
	EM CD4 T	17.70%	22.30%	27.86%	23.74%
	N CD4 T	52.56%	59.97%	67.26%	58.94%
	TEMRA CD4 T	3.61%	5.03%	6.30%	5.44%
	CD127- CD45RA+ CD4 T	1.01%	1.73%	2.49%	1.95%
	CD127+ CD45RA- CD4 T	25.02%	30.98%	39.04%	32.29%
	CD127+ CD45RA+ CD4 T	57.09%	63.50%	69.95%	62.44%
	CD45RA- Foxp3hi activated Treg	0.61%	1.33%	1.74%	1.33%
	CD45RA- FoxP3lo non Treg	1.43%	1.86%	2.57%	1.97%
	CD45RA+ FoxP3lo resting Treg	1.68%	2.36%	2.96%	2.58%
	CD27- CD28- CD4 T	0.02%	0.04%	0.27%	0.48%
	CD27- CD28+ CD4 T	2.83%	4.66%	6.52%	5.00%
	CD27+ CD28+ CD4 T	93.34%	95.24%	96.58%	94.38%
EM CD4 T cells	EM CD57+ CD4 T	3.70%	5.56%	7.16%	6.11%
	EM PD1+ CD4 T	10.14%	16.23%	20.21%	15.78%
	PD1+ CD57+ EM CD4 T	1.12%	1.51%	2.50%	2.00%
TEMRA CD4 T cellsT cells	TEMRA CD57+ CD4 T	5.40%	8.32%	13.07%	10.52%
CD8 T cells	EM CD8 T	21.18%	26.10%	32.56%	27.18%
	N CD8 T	38.50%	48.48%	57.69%	47.20%
	TEMRA CD8 T	16.05%	20.03%	27.97%	22.47%
	CD27- CD28- CD8 T	1.84%	4.35%	10.00%	7.50%
	CD27- CD28+ CD8 T	0.82%	1.41%	2.26%	2.11%
	CD27+ CD28- CD8 T	9.42%	14.33%	19.01%	15.85%
	CD27+ CD28+ CD8 T	68.04%	77.64%	84.29%	74.52%
EM CD8 T cells	EM CD57+ CD8 T	7.87%	12.49%	18.98%	14.63%
	EM PD1+ CD8 T	6.77%	12.08%	18.09%	14.67%
	PD1+ CD57+ EM CD8 TCD8 T	1.39%	2.44%	4.65%	3.59%
TEMRA CD8 T cells	TEMRA CD57+ CD8 T	12.88%	20.37%	34.62%	25.18%
CD27+ CD28+ CD8 T cells	CD27+ CD28+ CD69+ CD8 T	2.19%	3.24%	6.61%	5.95%
B cells	Double-negative-1 B	1.31%	1.66%	2.20%	1.92%
Double-negative-1 B	Double-negative-2 B	11.10%	17.72%	26.13%	20.34%
IgD+ CD27- B cells	IgM-low naïve B	60.51%	66.46%	70.19%	64.85%
	Naïve B	29.81%	33.54%	39.49%	35.15%
IgM+ IgD+ B cells	Unswitched mem B	1.91%	3.35%	4.97%	3.63%
CD27- IgM+ IgD+ B cellsB cells	Transitional B	7.47%	8.00%	8.71%	7.92%
IgM- IgD+ B cells	IgD only switched mem Bmem B	12.85%	18.44%	32.02%	23.01%
IgM- IgD- B cells	Early plasmablasts	1.50%	2.30%	3.05%	2.60%
	Plasmablasts	3.32%	4.80%	7.68%	5.85%
	Switched mem B	64.78%	69.39%	77.40%	70.13%
CD3- Lymphocytes	CD56^dim^ NK	30.64%	41.09%	49.35%	39.46%
	CD56^bright^ NK	2.33%	3.37%	4.40%	3.59%
CD56^dim^ NK	CD69+ CD56^dim^ NK	2.51%	3.48%	4.93%	5.06%
CD56^bright^ NK	CD69+ CD56^bright^ NK	24.82%	28.02%	31.18%	29.46%
CD3+ CD56+ T cells	CD8+ CD3+ CD56+ T	47.41%	55.59%	71.09%	58.48%
CD14- HLADR+ LIN-	mDCs	41.07%	53.63%	63.95%	52.21%
	pDCs	8.57%	12.41%	15.72%	12.79%
mDCs	CD16 mDCs	58.35%	66.47%	74.12%	65.13%
	CD1c CD16 mDCs	2.87%	5.12%	8.02%	5.57%
	CD1c mDCs	10.46%	16.66%	21.49%	17.44%
HLADR+ LIN-	classical monocytes	42.50%	62.02%	74.95%	59.16%
	intermediate monocytes	3.90%	8.21%	19.69%	12.58%
	non-classical monocytes	6.89%	10.02%	14.61%	10.76%

Cell frequencies (% of parent) measured by unsupervised clustering within the healthy children cohort (HC, n = 76).

**Figure 2:**
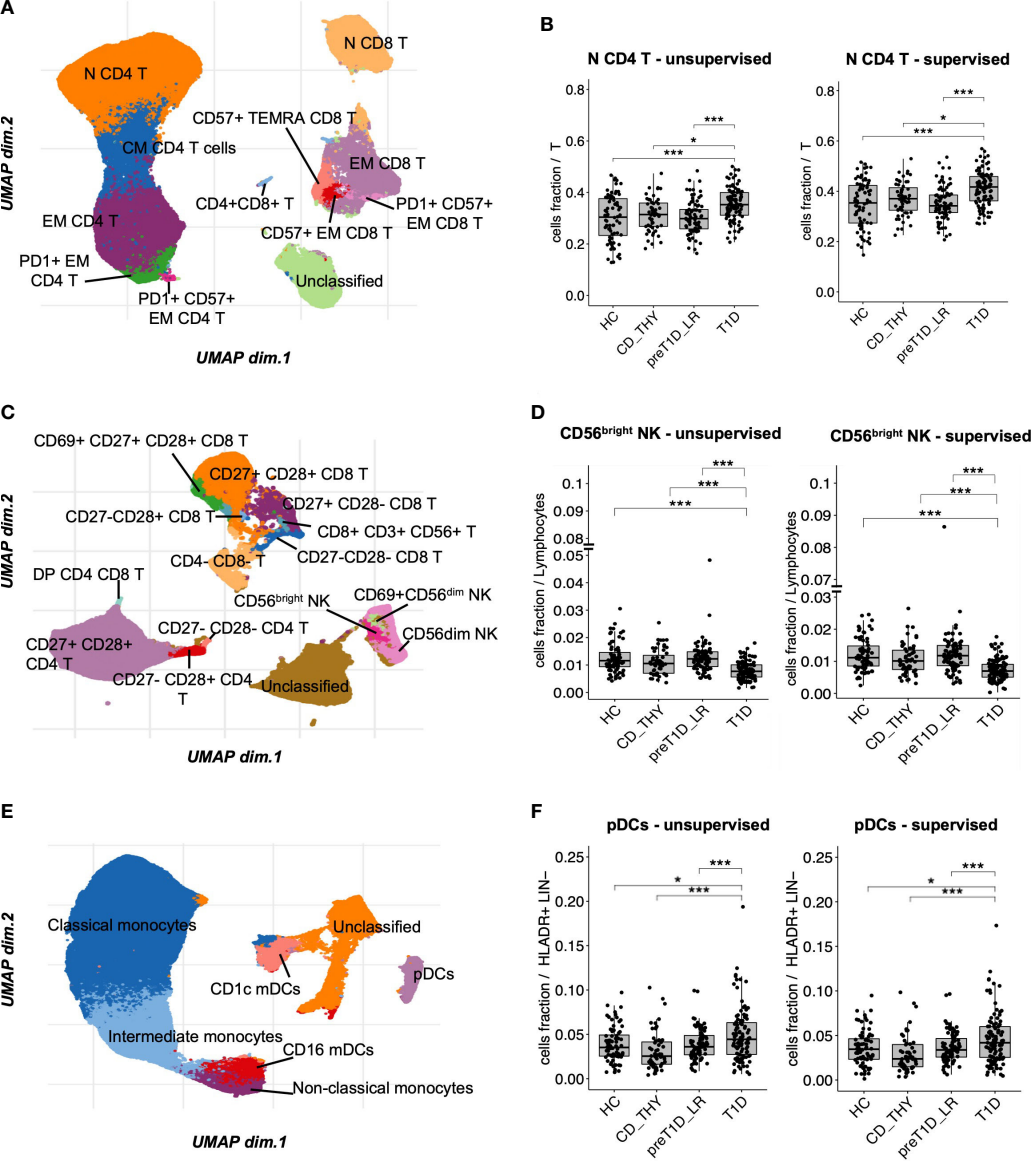
Unsupervised clustering shows increased frequency of N CD4 T cells and pDCs, and reduced frequency of CD56bright NK cells in T1D. UMAP data visualization of the cell clusters identified in the T cell **(A)**, T&NK cell **(C)** and DCs/monos **(E)** panels. Cell clusters differentially represented in T1D compared to all other groups in the T cell **(B)**, T&NK cell **(D)** and DC/mono **(F)** panels. HC, healthy controls; CD_THY, celiac or thyroid diseases; preT1D_LR, relatives with 0-1 autoantibodies; T1D, type 1 diabetes. Significant differences were determined by post hoc Tukey’s test performed on the linear regression model with age, sex, and technician as covariates (*p < 0.05, ***p < 0.001).

The T cell panel revealed 12 clusters (**Figure 2A** and **Supplementary Figure 2A**, bottom panel), with both unsupervised and semi-automated supervised analysis showing increased frequency of N CD4 T cells in T1D compared to the other groups (**Figure 2B**). The T&NK cell panel revealed 15 clusters (**Figure 2C** and **Supplementary Figure 2B**, bottom panel), showing a decreased frequency of CD56^bright^ NK cells specifically associated with T1D in both unsupervised and semi-automated supervised analysis (**Figure 2D**). The DCs/monos panel, which revealed 7 clusters (**Figure 2E** and **Supplementary Figure 2C**, bottom panel), showed an enrichment of plasmacytoid Dendritic Cells (pDCs) in T1D compared to other groups both using unsupervised and semi-automated supervised analysis (**Figure 2F**). The B cell panel revealed 11 clusters (**Supplementary Figure 2D**, bottom panel and **Supplementary Figure 6A**). Unsupervised analysis showed a specific reduction of transitional B cells in T1D, which was not confirmed by semi-automated supervised analysis in the comparison with pre-T1D_LR individuals (**Supplementary Figure 6B**). Finally, the Treg panel revealed 7 clusters (**Supplementary Figure 2E**, bottom panel); however, no differential representation of any of these clusters was found between the 4 groups. The frequency of all the remaining cell clusters identified with unsupervised analysis is shown in **Supplementary Figure 7**.

To conclude, unsupervised clustering, validated by a highly consistent semi-automated gating analysis, shows that the specific circulating immune profile of newly diagnosed type 1 diabetes is characterized by increased frequency of N CD4 T cells and pDCs, and decreased CD56^bright^ NK cells.

### Combined unsupervised and semi-automated supervised analyses show the circulating immune signature shared between type 1 diabetes and other autoimmune diseases

**Figure 3:**
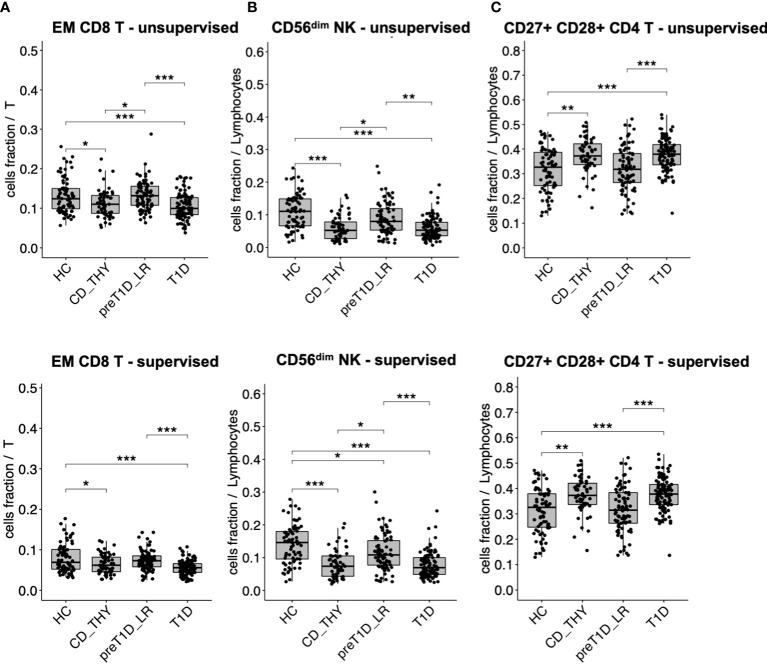
Cell populations altered in multiple autoimmune diseases. Cell subsets showing a reduced **(A, B)** or increased **(C)** frequency in the circulation of both patients with T1D and with celiac disease or autoimmune thyroiditis. HC, healthy controls; CD_THY, celiac or thyroid diseases; preT1D_LR, relatives with 0-1 autoantibodies; T1D, type 1 diabetes. Significant differences were determined by post hoc Tukey’s test performed on the linear regression model with age, sex, and technician as covariates (*p < 0.05, **p < 0.01, ***p < 0.001).

We found that specific cell clusters were consensually altered in both T1D and CD_THY as compared to HC and pre-T1D_LR, thus marking a feature of multiple autoimmune diseases rather than T1D. Unsupervised clustering showed a consensual reduction of EM CD8 T cells (**Figure 3A**) and CD56^dim^ NK cells (**Figure 3B**), accompanied by an increase of CD27^+^CD28^+^ CD4 T cells (**Figure 3C**) in both T1D and CD_THY, which was confirmed by semi-automated supervised analysis. These data allow the identification of a circulating immune signature not exclusively associated with type 1 diabetes, but rather shared by other autoimmune diseases such as celiac disease and autoimmune thyroiditis.

### T_R3-56_ cells are non-selectively reduced in newly diagnosed type 1 diabetes

**Figure 4:**
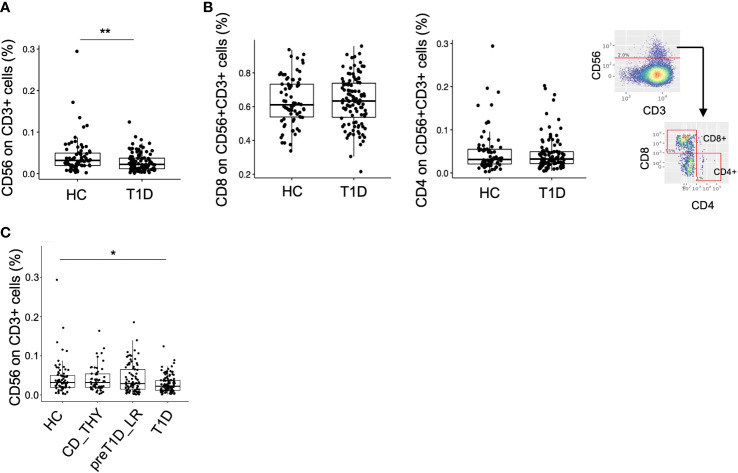
Validation of the TR3-56 cell biomarker in our cohort. Frequency of CD56^+^CD3^+^ cells (namely T_R3-56_ cells) in HC and T1D using the semi-automated gating strategy **(A)**. Frequency of CD8 and CD4 on TR_3-56_ cells and representative semi-automated gating strategy employed for the analysis. **(B)**. Frequency of T_R3-56_ cells in HC, CD_THY, preT1D_LR and T1D **(C)**. Significant differences were determined by Student’s t-test for pairwise comparisons and by Tukey *post-hoc* test on the linear regression model for multiple comparisons (*p < 0.05, **p < 0.01).

We leveraged our dataset as a tool to validate previously reported data by testing the frequency of CD3^+^CD56^+^ regulatory T cells (25), defined as T_R3-56_ cells, recently described to be reduced in patients with newly diagnosed type 1 diabetes compared to HC. Unsupervised analysis allowed the identification of 2 clusters co-expressing CD3 and CD56 (**Supplementary Figure 2B**, upper panel), one also expressing CD8, CD27 and CD28 and the other CD8 and CD27, which were merged in a single cluster called CD3^+^CD56^+^ (**Supplementary Figure 2B**, lower panel). No difference in the frequency of this cluster was evident between the 4 groups (**Supplementary Figure 7**). The semi-automated analysis, using the same gating strategy as in the original work (25), confirmed that the frequency of T_R3-56_ cells was reduced in T1D compared to HC also in our cohort, (**Figure 4A**), showing similar phenotypical characteristics, such as the expression level of CD4 and CD8 (**Figure 4B**). However, although differences between HC and T1D persisted when other control groups were included in the analysis, T_R3-56_ cells were found non-selectively decreased in type 1 diabetes as no differences could be detected with CD_THY or pre-T1D_LR (**Figure 4C**).

### Age and peripheral lymphocyte count, but not diabetic ketoacidosis, drive a slight segregation of the circulating immunome in newly diagnosed pediatric type 1 diabetes

**Figure 5:**
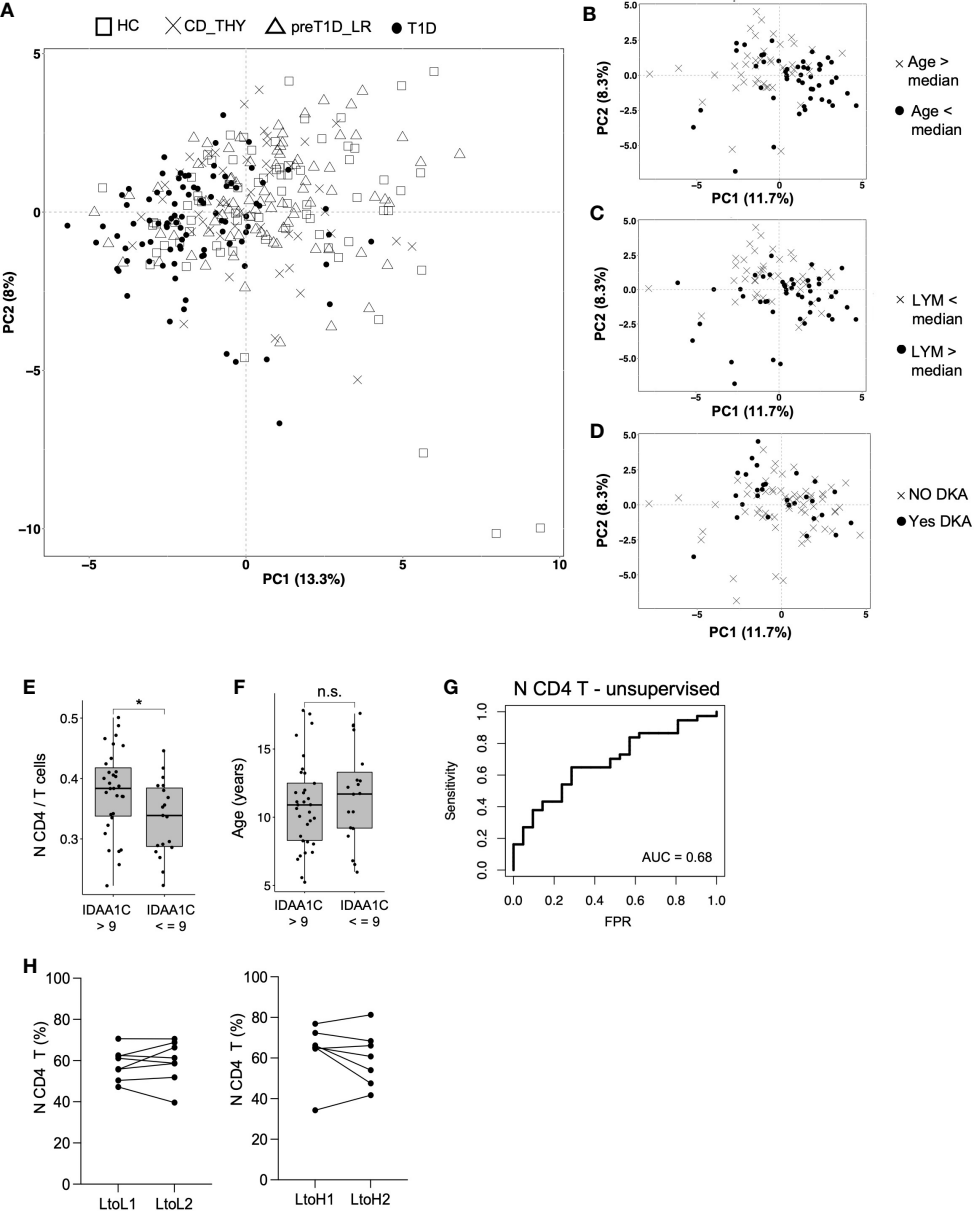
Frequency of N CD4 T cell at disease onset is associated with partial remission. Immunome-based principal component analysis performed on HC (n=73), CD_THY (n=47), preT1D-LR (n=82) and T1D (n=91) **(A)**; segregation of the circulating immunome of newly diagnosed T1D patients (n=91) based on median age **(B)**, median peripheral lymphocyte count **(C)** and presence of DKA **(D)**; comparison of the frequency of N CD4 T cell **(E)** and age **(F)** between newly diagnosed T1D patients with IDAA1C >9 (n=39) and IDAA1C ≤9 (n=21) at the 12-month follow-up visit; model for the prediction of partial remission based on the frequency N CD4 T cell at disease diagnosis **(G)**; N CD4 T cell frequency in relatives who maintained a stable Stage 0 status or during the transition from Stage 0 (0-1 Aab) to a Stage≥1 condition (≥2Aabs) **(H)**. LYM, lymphocytes; DKA, diabetic ketoacidosis; IDAA1c, insulin dose adjusted HbA1c; AUC, Area under the curve: N CD4 T cells: naïve CD4 T cells. Significant differences were determined by unpaired (4E, F) and paired (4H) Student’s t-test for pairwise comparisons (*p < 0.05, ns, not significant.

As we expected to observe a segregation of the circulating immunome of T1D patients compared to other groups, we performed a principal component analysis (PCA) (24) on the 46 detected clusters. Patients with T1D showed a weak segregation from other groups (which was more evident in the PC 1) explaining only 13% of the total variance (**Figure 5A**). Then, we tested whether, in newly diagnosed T1D patients, the following variables could affect the variance of the circulating immunome: age, sex, BMI percentile, type of Aab, white blood cell count, number of lymphocytes, monocytes and neutrophils, presence of diabetic ketoacidosis (DKA), family history of type 1 diabetes or other autoimmune diseases and presence of HLA-DR3 and/or DR4. We found that age (> or < the median, i.e., 11.0 years, **Figure 5B**) and lymphocyte count (> or < the median, i.e., 2.6 ×10³ cells/μL, **Figure 5C**), but not DKA (**Figure 5D**) or any the other tested parameters (data not shown), drove a slight, although evident, segregation of the circulating immunome.

### Frequency of N CD4 T cells at disease onset is associated with partial remission

To determine whether immune cell subsets found to be altered in newly diagnosed T1D may have a pathogenic potential, we explored their relationship with partial remission. The IDAA1c estimates residual beta cell function, with IDAA1C ≤9 considered as surrogate marker of partial remission. We found that the frequency of N CD4 T cells, but not pDC or CD56^bright^ NK cells, was increased at disease onset in individuals with IDAA1C >9 at the 12-month follow-up visit compared to those with IDAA1C ≤9 (**Figure 4E**). Notably, this was independent of age, as the age range of T1D patients with IDAA1C ≤9 vs. >9 was similar (**Figure 4F**). We also found that N CD4 T cells show the ability, albeit mild, to predict partial remission (AUC 0.68), indicating a potential biological relevance in symptomatic pediatric type 1 diabetes (**Figure 4G**). To test the possible involvement of N CD4 T cells in early disease stages, their frequency was tested in relatives of patients with type 1 diabetes who transitioned from Stage 0 (at T0) to Stage≥1 (at T1) and in a cohort of age and sex-matched relatives who maintained the Stage 0 status both at T0 and T1. No difference in the time span between T0 and T1 was observed between the two groups of relatives (i.e., mean of 2.5 years for Stage 0-T0 to Stage 0-T1 vs. mean of 2.7 years for Stage 0-T0 to Stage ≥1-T1, *ns*). Supervised analysis showed that the transition through pre-symptomatic stages of type 1 diabetes is not associated with a change in the frequency of N CD4 T cells (**Figure 4H**), indicating that this cell subset may be a player of the late phase of the autoimmune response associated with type 1 diabetes.

## Discussion

In this study we unbiasedly identified the circulating immune fingerprint of pediatric patients with newly diagnosed type 1 diabetes compared to different control groups, including healthy controls, relatives with a low-risk of developing type 1 diabetes and patients with celiac disease and thyroiditis. We employed one of the most advanced (26), widely used (27), unsupervised clustering technique for the analysis of multiparameter flow cytometry data and validated the results with a fast and reproducible semi-automated gating method (26). Indeed, the pipelined semi-automated supervised analysis implemented in this study was highly reliable in reproducing results of the unsupervised clustering (r=0.94) for the vast majority of the cell populations–including rare cell subsets. The poorest correlations were observed in cell populations expressed as a smear, without a clear-cut discrimination between the negative and the positive edge. The interpretation of markers expressed as a continuous is subtle in flow cytometry data analysis and clustering machine-learning-based algorithms are in development to address this issue (28).

Three immune cell subsets, i.e. N CD4 T cells, pDCs and CD56^bright^ NK cells, were found differentially represented between new-onset type 1 diabetes and all other groups in both the analysis. Increased circulating frequency of N CD4 T cells has already been described by previous reports (8, 12); noteworthy, N CD4 T cells have been shown to be heterogeneous in phenotype, function, dynamics, gene expression profile and differentiation status, covering a whole spectrum of cells with different properties (29). Indeed, it is well-established that the major genetic determinants of type 1 diabetes risk are polymorphisms of class II HLA genes encoding DQ and DR (30), thus indicating a leading contribution of the interactions between CD4 T cells and antigen presenting cells; our results suggest that the phenotypic and functional characterization of N CD4 T cells would allow the identification of candidate subsets with potential pathogenicity in the context of human type 1 diabetes. This hypothesis was further corroborated by the evidence that the frequency of N CD4 T cells at disease onset was associated with partial remission, independently of age, confirming, in a broader cohort, the observations of a recent report (31), and others associating the frequency of CD4 memory T cells with a longer partial remission (32). However, N CD4 T cell frequency was found unaltered in pre-symptomatic stages of type 1 diabetes, suggesting that the role of these cells may be crucial in a later phase of the autoimmune response.

Plasmacytoid DCs are mainly recognized for their swift and massive production of type I interferon (33) and are capable of activating several cell subsets, such as CD8 cytotoxic T cells and regulatory T cells (34, 35), and of stimulating B-cell activation, differentiation into plasma cells, and antibody production (36, 37). Our data confirm previous observations showing that the balance of peripheral dendritic cells is deeply altered at diabetes diagnosis, with a markedly elevated proportion of pDCs and reduction of mDCs compared with control subjects (38), and that pDCs are not increased in other autoimmune diseases (39, 40).

Although a small but distinct reduction in NK cell frequency has previously been found both in patients with other autoimmune diseases (41, 42) and with recent-onset diabetes (10), no differences were described in the repartition of the CD56^high^/CD16^-^ and CD56^dim^/CD16^+^ NK cell subsets (10). Here, we show a selective decrease of the CD56^bright^ NK cell subset in newly diagnosed pediatric type 1 diabetes. The distribution of immune cells in peripheral blood tells only part of the story, as resident cell subsets located in the pancreas are the relevant players in the pathogenesis of type 1 diabetes. However, these changes in the blood could reflect similar alterations in the tissue or could be the result of a relative sequestration/migration of specific populations into the pancreas. This may be the case of CD56^bright^ NK cells, which are known to express high levels of CCR7 and CXCR3—thus preferentially migrating to secondary lymphoid organs—and to produce very high levels of cytokines (43). An increased homing to lymph nodes, with high local production of cytokines, and a consequent decrease in the peripheral blood of patients with new-onset type 1 diabetes could be hypothesized.

Besides validation of previously described data and discovery of new cellular biomarkers, our dataset clarified previous contrasting reports on specific cell subtypes. As an example, CD45RA^+^ Tregs had been described as both normal (14) or altered (15) in type 1 diabetes; in our study, a normal frequency of this cell subpopulation was observed in newly diagnosed type 1 diabetes. In addition, the implemented pipeline of unbiased analyses, combined with the comparison with 3 control groups, allowed to elucidate how some of the previously reported type 1 diabetes-related immune alterations, such as a reduction of EM CD8 T (8) and CD56^dim^ NK cells (44), are rather markers shared with other autoimmune diseases, or are not selectively altered in type 1 diabetes, as in the case of T_R3-56_ cells (25). Therefore, the analytic approach proposed in this article appears to be robust, and our large cohort has the potential to be used by other investigators as a reference dataset.

Finally, we found that the peripheral immune profile could not discriminate patients with new-onset type 1 diabetes from other clinical groups, indicating that the non-(antigen)specific peripheral immune system is presumably inadequate for an exhaustive discrimination between individuals or diseases. However, age and lymphocyte count had an effect, although mild, on the segregation of the circulating immunome of patients with type 1 diabetes, confirming a recent study showing that a higher lymphocyte count is evident in younger patients with type 1 diabetes (13). Unexpectedly (45), DKA did not appear to have a major effect on the profile of the peripheral immunome, and this is likely due to the 5-to-10-day interval from diagnosis that we observe before the blood sample collection.

The extensive pediatric cohort of individuals, the strong consistency between unbiased analyses, and the proper assortment of different control groups are major strengths of our work. Nevertheless, although comprehensive for the well-defined lymphoid and myeloid markers, a limitation of this study is that we used five separate antibody panels, mainly comprised of lineage markers, which limits the chance to detect unconventional cell subsets. Furthermore, this work lacks an internal validation cohort, which would have allowed the identification and elimination of further possible biases.

This study provides an unbiased and reproducible characterization of the circulating immune cell profile uniquely associated with new-onset type 1 diabetes in a large pediatric cohort, representing a reference dataset to be exploited for validation or discovery purposes to elucidate the pathogenesis of type 1 diabetes.

## Data availability statement

The original contributions presented in the study are included in the article/**Supplementary Materials**. Further inquiries can be directed to the corresponding author.

## Ethics statement

The studies involving human participants were reviewed and approved by Ethics Committee Ospedale San Raffaele (protocols TIGET004-DRI003 and NHPROT32803-TN01). Written informed consent to participate in this study was provided by the participants’ legal guardian/next of kin.

## Author contributions

CBG and EB planned and performed the analyses, contributed to data interpretation and discussion, and drafted, revised, and approved the final version of the article. DC, AM, and VC performed data acquisition and analysis, and revised and approved the final version of the article. AS, VI, and FR performed sample storage and data management, and revised and approved the final version of the article. EM, VC, and VF provided biological samples, performed data acquisition, patient selections, contributed to data interpretation, and revised and approved the final version of the article. SDR and BAM performed data acquisition and analysis, contributed to data interpretation, and revised and approved the final version of the article. GMS supervised the statistical analysis, contributed to data interpretation, and revised and approved the final version of the article. DPM, AG, LP, EB, MB, MJM, and RB contributed to patient selection, provided biological samples, contributed to data interpretation, and revised and approved the final version of the article. AP designed and coordinated the study, interpretated the data, and drafted and revised the final version of the article. All authors contributed to the article and approved the submitted version.

## Funding

This study was funded by the Italian Ministry of Health – Ricerca Finalizzata RF-2016-02364070. This study was funded by the Italian Ministry of Health – Ricerca Finalizzata RF-2016-02364070.

## Acknowledgments

AP is supported by the Juvenile Diabetes Research Foundation JDRF (n. 3-APF-2019-744-AN) and Fondazione Italiana Diabete.

## Conflict of interest

The authors declare that the research was conducted in the absence of any commercial or financial relationships that could be construed as a potential conflict of interest.

## Publisher’s note

All claims expressed in this article are solely those of the authors and do not necessarily represent those of their affiliated organizations, or those of the publisher, the editors and the reviewers. Any product that may be evaluated in this article, or claim that may be made by its manufacturer, is not guaranteed or endorsed by the publisher.
