# Supplementary material for: Combined unsupervised and semi-automated supervised analysis of flow cytometry data reveals cellular fingerprint associated with newly diagnosed pediatric type 1 diabetes
Source: Front Immunol. 2022 Oct 27;13:1026416. doi: 10.3389/fimmu.2022.1026416 (PMC9647173; doi:10.3389/fimmu.2022.1026416)
Supplement: Supplementary file 1 [file DataSheet_1.pdf]

Supplementary Material

Supplementary Data

Preliminary set up for flow cytometry analysis

Unstained cells with different PMT voltages were acquired starting from 0 (for all channel) up to 800 increasing by 50. These samples were used to evaluate the electronic noise and the minimum voltage. Rainbow beads with different PMT voltages were acquired, increasing by 20, to evaluate PMT linearity and assess if the brightest populations were within the linear range of the detector. A set of fluorescence minus one (FMO) control stainings were included in each panel. These FMO control stainings were used to establish gates for PD-1 and CD57 in the T cell panel, CD27 in the T+NK cell panel, CD1c and CD16 in the DC panel, CD27, CD24 and CD38 in the B cell panel, and FoxP3 in the Treg panel.

Unsupervised clustering

Individual .fcs files were imported and pre-processed in R environment (version 4.0.3). Spillover coefficients were further optimized for each fluorophore using single-color controls with Autospill web tool https://autospill.vib.be/public/ - /run (1). The spillover matrices were imported in R environment using *cyto_compensate()* function of CytoExploreR package (version 1.0.8) (2). Channels were logically transformed using cyto_transform() function (2), debris and doublets were removed and a standard gating strategy was performed using the OpenCyto package (version 2.2.0) (3). T cells (CD3+), lymphocytes, CD4 T cells (CD4+), B cells (CD19+) and HLADR+LIN- were extracted to perform the unsupervised clustering of T cells, T&NK cells, Tregs, B cells, and DCs/monos panel, respectively. Unsupervised analysis and representation of cell populations was realized with FlowSOM algorithm (4) included in the CyTOF/CATALYST pipeline (version 1.14.1) (5), as well as the visual representations of cell populations. The FlowSOM algorithm is extremely fast as it makes use of self-organizing maps (6) and outperformed other methods for the analysis of single-cell data (7). In particular, marker intensities were reverse transformed to restore the data to its original format and the inverse hyperbolic sine transformation with cofactor 150 was applied to the raw signal intensities. Moreover, only non-lineage markers were used to perform the clustering. The FlowSOM algorithm was run with default parameters and an overestimated initial number of clusters to avoid the loss of rare cell subtypes, as suggested by the authors of the method (8). Clusters showing similar marker expression profiles were merged after a careful evaluation of the Uniform Manifold Approximation and Projection (UMAP) and the dendrogram of hierarchical clustering, as depicted in Supplementary Figure 2 A-E upper panels. Collectively we identified 52 final clusters, including 5 unclassified clusters and 1 duplicate. Next, we applied the non-linear dimensionality reduction technique UMAP to the lineage marker levels, selecting a maximum of 500 cells per sample, using the *runDR()* function included in CyTOF workflow included in CyTOF workflow, for visualization of high-dimensional data (9). Cells were colored according to their FlowSOM cluster membership. Clusters identifying unknown cell types were included in the heatmap and UMAP but excluded from the subsequent analysis.

Semi-automated supervised gating

To validate unsupervised clustering results, and to minimize manual biases, we set up a semi-automated supervised gating strategy using the OpenCyto R package (3) (gating strategies are shown

in Supplementary Figures 3-4): (i) compensations were first adjusted using the Autospill algorithm (1); (ii) data were transformed into Logical Scale; (iii) to adjust for between-sample and batch variation, all markers satisfying the rules for normalization were subject to the landmark alignment procedure (10), using the normalize() function in the flowStats package (version 3.40.1); (iv) the supervised gating strategy was performed using different auto-gating

functions (3); (v) possible outliers were detected as having a median z-score > 3. They were manually checked one by one and either left untouched if the population was properly delineated, discarded if staining failed due to lack of antibodies, or manually corrected if the semi-automated gating failed to properly detect the cell population. Briefly, CD3+, CD19+ and Lin-HLADR+ were used to determine pan T, pan B and DCs/monocytes, respectively. NK cells were identified within the CD3− population based on CD56 expression. Within the T cell population, CD4 and CD8 T cells were identified. For CD4 and CD8 T cells, naive, central memory, effector memory, and terminal effector memory subpopulations were identified based on CD45RA and CCR7 expression. CD57 and PD1 were checked on CD8 and CD4 T cells subpopulations. For CD4 T cells, CD45RA- FoxP3hi activated Treg cells were determined based on FoxP3+++ CD25+++ CD45RA- expression, and CD45RA+ FoxP3lo resting Treg cells as FoxP3+ CD25+ CD45RA+. Subpopulations of NK cells were determined based on the brightness of CD56. CD69 was checked on CD8 T cells, CD4 T cells and NK cells. Within the B cell population, naive and memory B cells were identified based on CD27 expression. Other subpopulations were identified based on the relative expression of IgD, IgM, CD21, CD24, CD38. Finally, HLADR+ cells were separated into monocytes, further subclassified based on CD16 and CD14 expression, and DCs, defined as CD14−, where DC subsets were determined by CD11c (myeloid) and CD123 (plasmacytoid) expression. Myeloid DCs subpopulations were determined based on CD1c and CD16 expression. In the longitudinal analysis of relatives who persisted in Stage 0 or transitioned to Stage ≥1 type 1 diabetes, a manual gating strategy was performed.

Statistics

Fractions of each cell population revealed by unsupervised and supervised semi-automated analysis were transformed using bestNormalize R package (version 1.8.2) (11) to fit a normal distribution, before comparing between the four groups.

References

1. Roca, CP, Burton, OT, Gergelits V, Prezzemolo T, Whyte CE, Halpert R, Kreft L, Collier J, Botzki A, Spidlen J, Humblet-Baron S, Adrian Liston A. AutoSpill is a principled framework that simplifies the analysis of multichromatic flow cytometry data. Nat Commun (2021) 12, 2890. doi: 10.1038/s41467-021-23126-8.

2. CytoExploreR: Interactive Analysis of Cytometry Data. Dillon Hammill (2021). R package version 1.1.0.

3. Finak G, Frelinger J, Jiang W, Newell EW, Ramey J, Davis MM, Kalams SA, De Rosa SC, Gottardo R. OpenCyto: an open source infrastructure for scalable, robust, reproducible, and automated, end-to-end flow cytometry data analysis. PLoS Comput Biol (2014) 10(8):e1003806. doi:10.1371/journal.pcbi.1003806.

4. Van Gassen S, Callebaut B, Van Helden MJ, Lambrecht BN, Demeester P, Dhaene T, Saeys Y. FlowSOM: Using self-organizing maps for visualization and interpretation of cytometry data. Cytometry A (2015) 87(7):636-45. doi: 10.1002/cyto.a.22625.

5. Crowell HL, Chevrier S, Jacobs A, Sivapatham S, Tumor Profiler Consortium, Bodenmiller B, Mark D. Robinson MD. An R-based reproducible and user-friendly preprocessing pipeline for CyTOF data. F1000Res. (2020) 9, 1263.

6. Saeys, Y; Gassen SV; Lambrecht BN. Computational flow cytometry: helping to make sense of high-dimensIonal immunology data. Nat. Rev. Immunol (2016). 16:449–462.

7. Weber, L. M. & Robinson, M. D. Comparison of clustering methods for high-dimensional single-cell flow and mass cytometry data. Cytometry A (2016) 89:1084–1096.

8. Quintelier K, Couckuyt A, Emmaneel, A, Aerts J, Saeys Y, Van Gassen S. Analyzing high-dimensional cytometry data using FlowSOM. Nat Protoc (2021) 16:3775–3801. doi:10.1038/s41596-021-00550-0.

9. McInnes L, Healy J, Melville J. UMAP: Uniform Manifold Approximation and Projection. 2018. arXiv:1802.03426 [stat.ML]. doi: 10.48550/arXiv.1802.03426.

10. Finak G, Jiang W, Krouse K, Wei C, Sanz I, Phippard D, Asare A, De Rosa SC, Steve Self S, Gottardo R.High Throughput Flow Cytometry Data Normalization for Clinical Trials, Cytometry A (2014) 85(3): 277–286. doi: 10.1002/cyto.a.22433

11. Peterson RA, Cavanaugh JE. “Ordered quantile normalization: a semiparametric transformation built for the cross-validation era”. Journal of Applied Statistics (2019) 1-16. doi: 10.1080/02664763.2019.1630372.

Supplementary Tables

Marker	Fluorophore	µl/in 100 µl	Clone	Company	Cat. no.
T cell panel					
CCR7	PE	2	G043H7	Biolegend	353204
CD3	PerCP	2	SK7	Biolegend	344814
CD4	APC	2	SK3	Biolegend	344614
CD45	BrilliantViolet510	2	H130	Biolegend	304036
CD45RA	FITC	2	HI100	Biolegend	304148
CD57	Pacific Blue	2	HCD57	Biolegend	322315
CD8	APC/Cy7	2	SK1	Biolegend	344714
PD-1	PECy7	2	J105	eBioscience	25-2799-42
T&NK cell panel					
CD45	BrilliantViolet510	2	H130	Biolegend	304036
CD3	PerCP	2	SK7	Biolegend	344814
CD4	Pacific Blue	2	RPA-T4	Biolegend	300521
CD69	APC	2	FN50	Biolegend	310918
CD56	PE	2	HCD56	Biolegend	318305
CD8	PECy7	2	RPA-T8	Biolegend	301012
CD27	APC/Cy7	2	O323	Biolegend	302816
CD28	AlexaFluor488	2	CD28.2	Biolegend	302916
Treg panel					
CD25	APC	2	2A3	BD	340907
CD3	PerCP	2	SK7	Biolegend	344814
CD4	Pacific Blue	2	RPA-T4	Biolegend	300521
CD45	BrilliantViolet510	2	H130	Biolegend	304036
CD127	PECy7	2	R34.34	Beckman	A64618
CD45RA	PE	2	HI100	Biolegend	304108
FoxP3	Alexa488	4	259D	Biolegend	320212
B cell panel					
CD19	BrilliantViolet421	2	HIB19	Biolegend	302234
CD21	PECy7	2	Bu32	Biolegend	354911
CD24	APC	2	MLS	Biolegend	311117
CD45	BrilliantViolet510	2	H130	Biolegend	304036
CD27	APC/Cy7	2	O323	Biolegend	302816
CD38	PerCP	2	HIT2	Biolegend	303519
IgD	AlexaFluor488	2	IA6-2	Biolegend	348216
IgM	PE	2	MHM-88	Biolegend	314508
DCs/monos					
LIN	APC	2	UCHT1,HCD14, HIB19,2H7, HCD56	Biolegend	348703

HLADR	APC-CY7	2	L243	Biolegend	307618
CD14	AlexaFluor488	2	HCD14	Biolegend	325610
CD45	BrilliantViolet510	2	H130	Biolegend	304036
CD1c	PerCP	2	L161	Biolegend	331511
CD16	BrilliantViolet421	2	3G8	Biolegend	302038
CD123	PECy7	2	6H6	Biolegend	306009
CD11c	PE	2	Bu15	Biolegend	337205

Supplementary Table 1. Antibodies used in the 5 FACS panels

Supplementary Figures

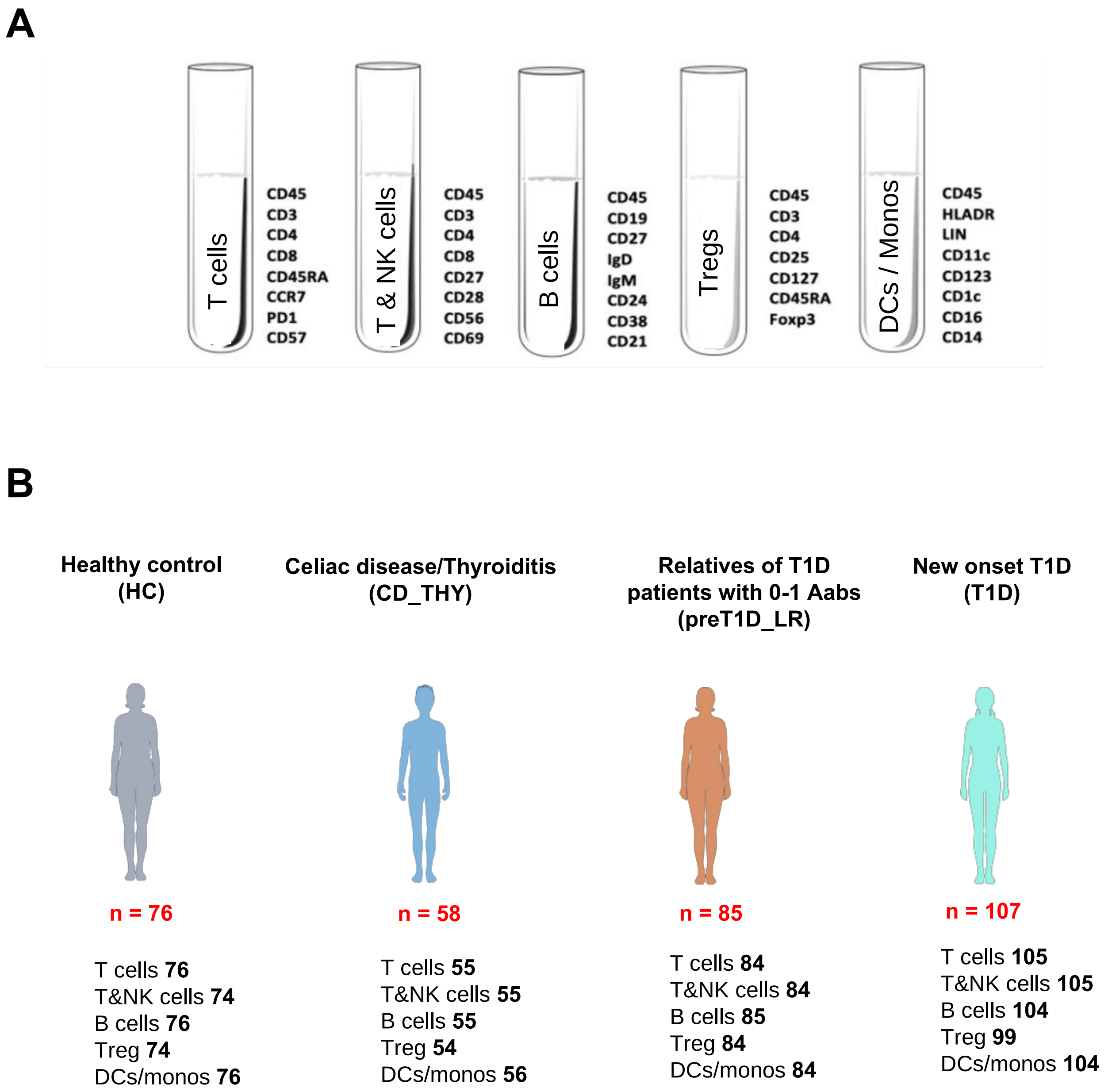

Supplementary Figure 1. FACS panels and study cohort. Markers used in the T cells, T+NK cells, B cells, Regulatory T cells, Dendritic cells panel, respectively (A). Subjects enrolled in the study and details about the number of panels performed per group (B).

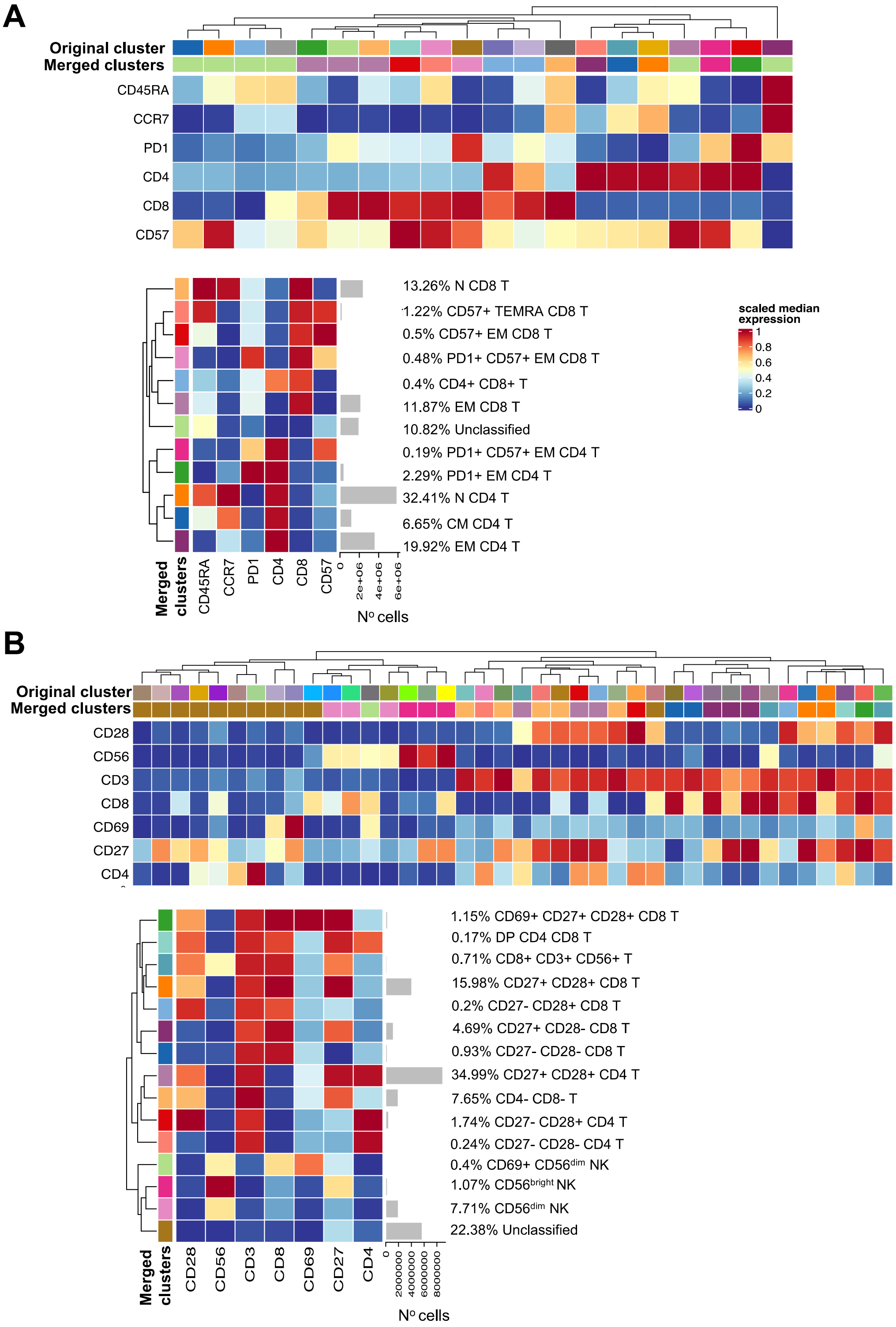
A
Original cluster
Merged clusters
CD45RA
CCR7
PD1
CD4
CD8
CD57
13.26% N CD8 T
1.22% CD57+ TEMRA CD8 T
0.5% CD57+ EM CD8 T
0.48% PD1+ CD57+ EM CD8 T
0.4% CD4+ CD8+ T
11.87% EM CD8 T
10.82% Unclassified
0.19% PD1+ CD57+ EM CD4 T
2.29% PD1+ EM CD4 T
32.41% N CD4 T
6.65% CM CD4 T
19.92% EM CD4 T
scaled median expression
1
0.8
0.6
0.4
0.2
0
Merged clusters
0
2e+06
4e+06
6e+06
Nº cells
B
Original cluster
Merged clusters
CD28
CD56
CD3
CD8
CD69
CD27
CD4
1.15% CD69+ CD27+ CD28+ CD8 T
0.17% DP CD4 CD8 T
0.71% CD8+ CD3+ CD56+ T
15.98% CD27+ CD28+ CD8 T
0.2% CD27- CD28+ CD8 T
4.69% CD27+ CD28- CD8 T
0.93% CD27- CD28- CD8 T
34.99% CD27+ CD28+ CD4 T
7.65% CD4- CD8- T
1.74% CD27- CD28+ CD4 T
0.24% CD27- CD28- CD4 T
0.4% CD69+ CD56dim NK
1.07% CD56bright NK
7.71% CD56dim NK
22.38% Unclassified
Merged clusters
0
2000000
4000000
6000000
8000000
Nº cells

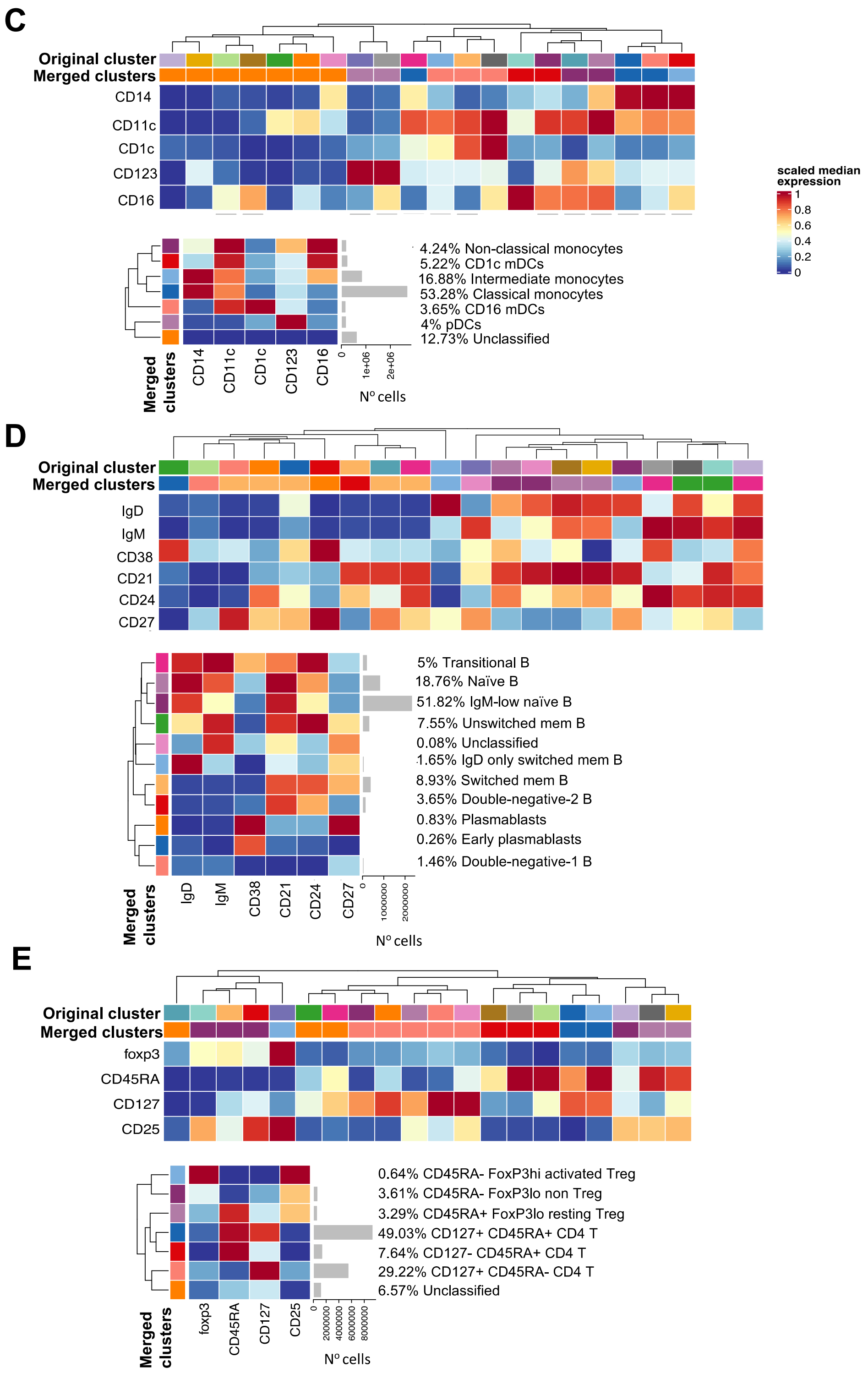

Supplementary Figure 2 Merging strategies of unsupervised analysis of flow cytometry data. Heatmaps showing markers expression in each cluster. Merging strategies used to identify the final clusters in the T cells (A), T&NK cells (B), B cells (C), Regulatory T cells (D), DC/mono panels (E).

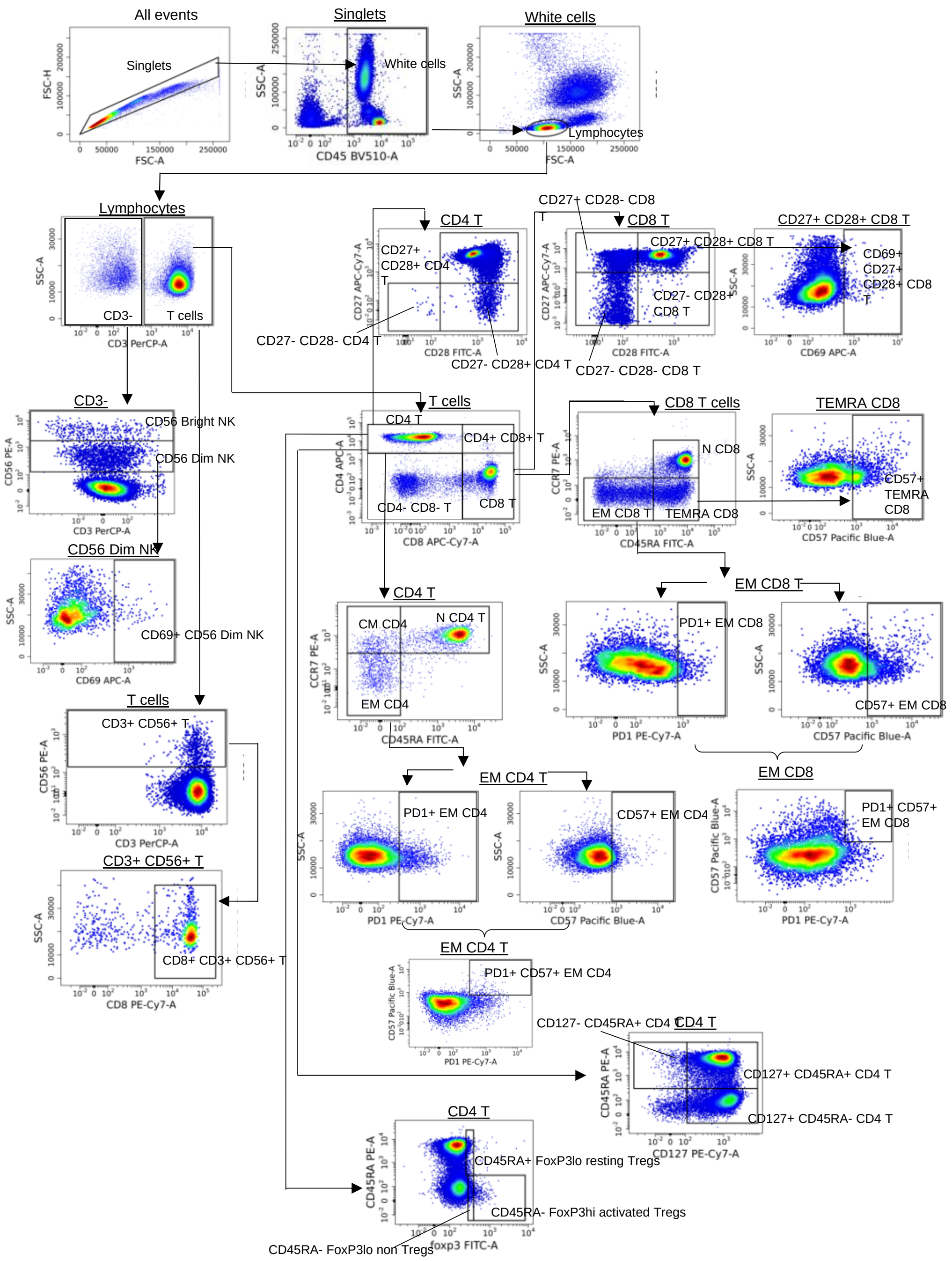

Supplementary Figure 3. Semi-automated gating strategy for the T, T&NK and Treg panels. After singlets removal, CD45 was used to identify white blood cells and a morphological gate was set; CD3 was then used to determine pan T. NK cells were identified within the CD3− population based on CD56 expression. Within the T cell population, CD4 and CD8 T cells were identified. For CD4 and CD8 T cells, naive, central memory, effector memory, and terminal effector memory subpopulations were identified based on CD45RA and CCR7 expression. CD57 and PD1 were checked on CD8 and CD4 T cells subpopulations. For CD4 T cells, CD45RA- FoxP3hi activated Treg cells were determined based on FoxP3+++ CD25+++ CD45RA- expression, and CD45RA+ FoxP3lo resting Treg cells as FoxP3+ CD25+ CD45RA+. Subpopulations of NK cells were determined based on the brightness of CD56. CD69 was checked on CD8 T cells, CD4 T cells and NK cells.

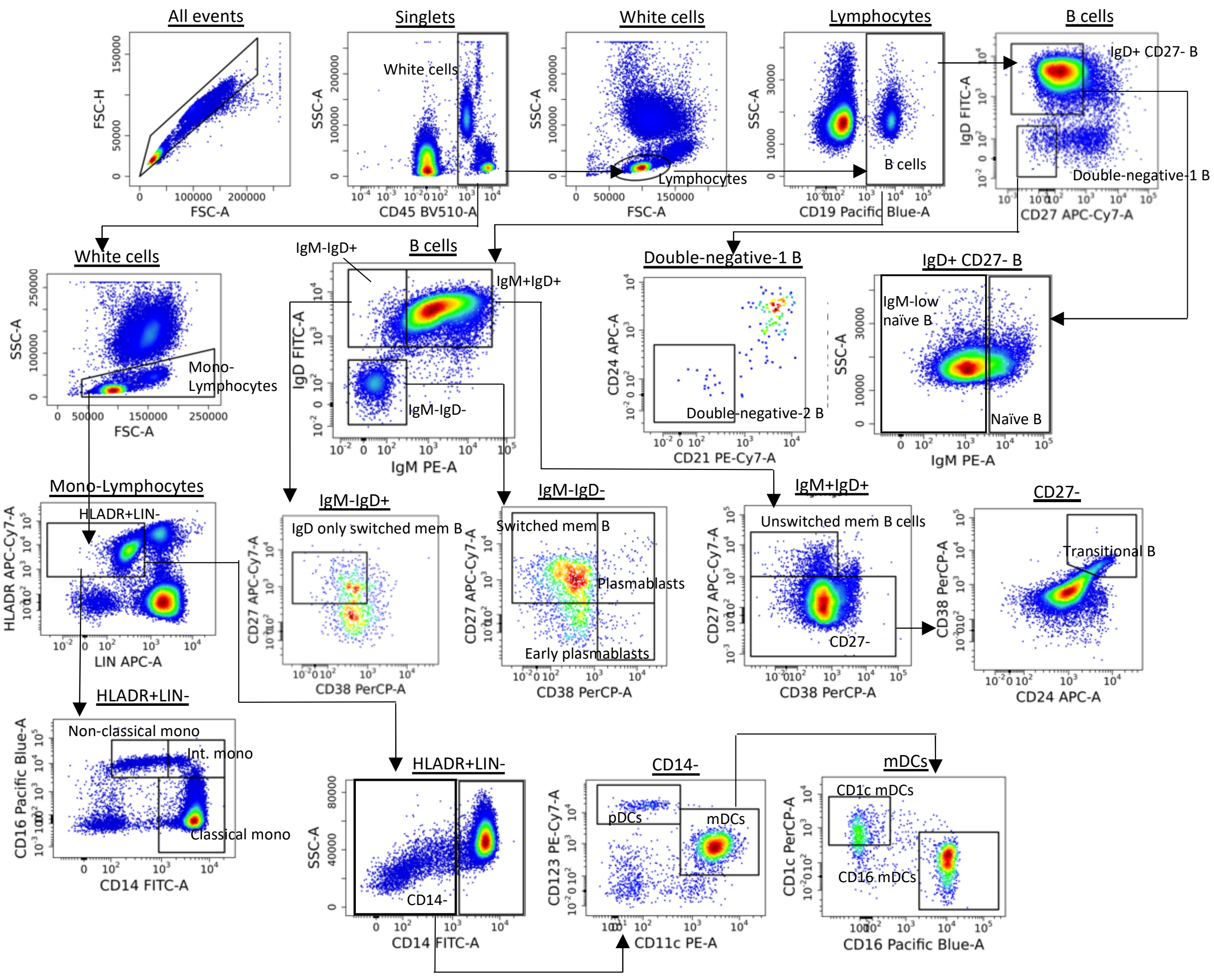

Supplementary Figure 4. Semi-automated gating strategy for the B cells and DCs/monos panels. After singlets removal, CD45 was used to identify white blood cells and a morphological gate was set; CD19 and Lin-HLADR+ were then used to determine pan B and DCs/monocytes, respectively. Within the B cell population, naive and memory B cells were identified based on CD27 expression. Other subpopulations were identified based on the relative expression of IgD, IgM, CD21, CD24, CD38. HLADR+ cells were separated into monocytes, further subclassified based on CD16 and CD14 expression, and DCs, defined as CD14−, where DC subsets were determined by CD11c (myeloid) and CD123 (plasmacytoid) expression. Myeloid DCs subpopulations were determined based on CD1c and CD16 expression.

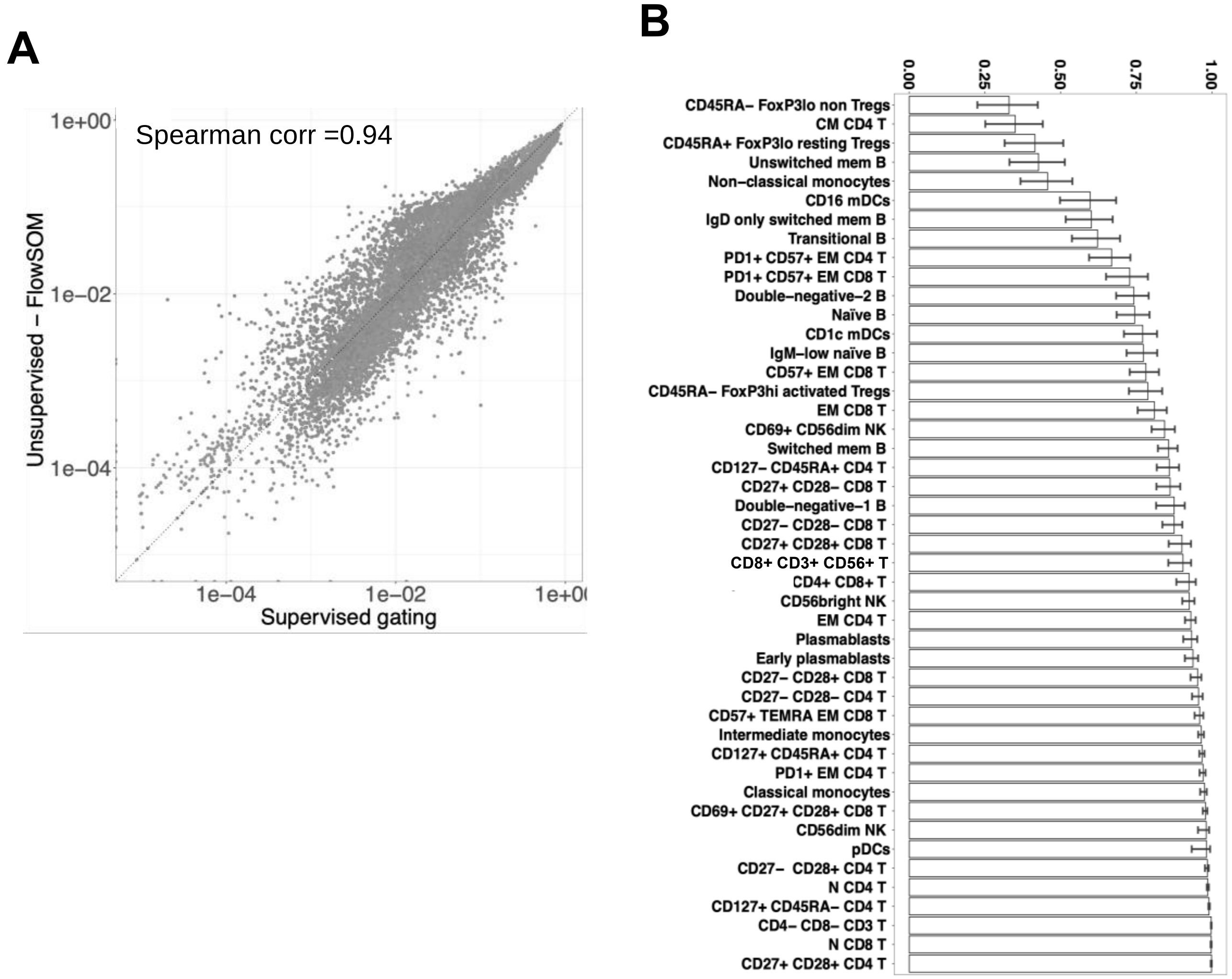

Supplementary Figure 5. Correlation between unsupervised and semi-automated supervised analysis. Overall (A) and cell subset-specific (B) Spearman correlation between the frequency of cell populations assessed through the unsupervised and semi-automated supervised analysis.

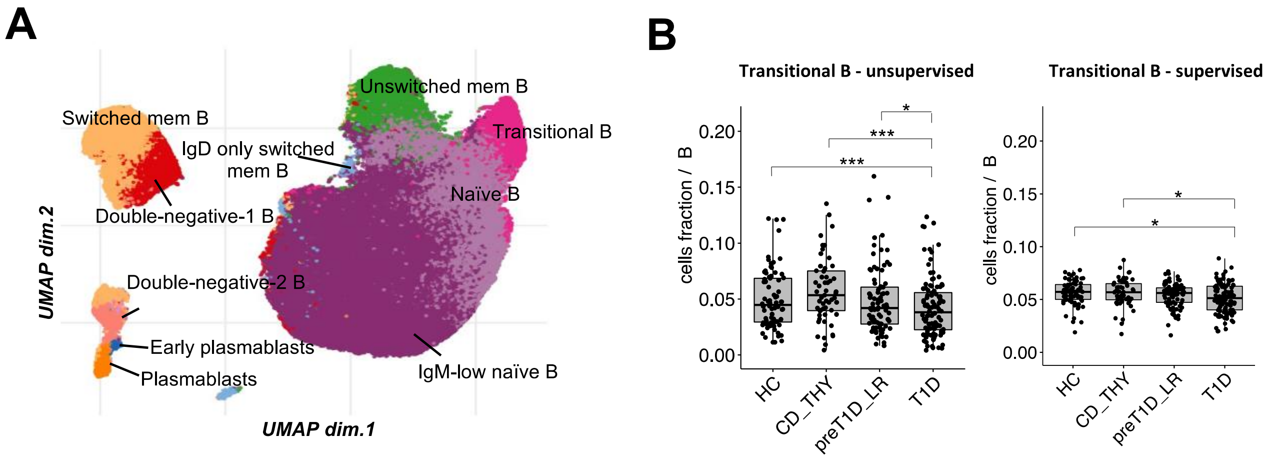

Supplementary Figure 6. Unsupervised clustering of the B cell panel. UMAP data visualization of the cell clusters identified in the B cell panel (A). Cell population differentially represented in T1D compared to all other groups. HC, healthy controls; CD_THY, celiac or thyroid diseases; preT1D_LR, relatives with 0-1 autoantibodies; T1D, type 1 diabetes. * $p < 0.05$, ** $p < 0.01$, *** $p < 0.001$.

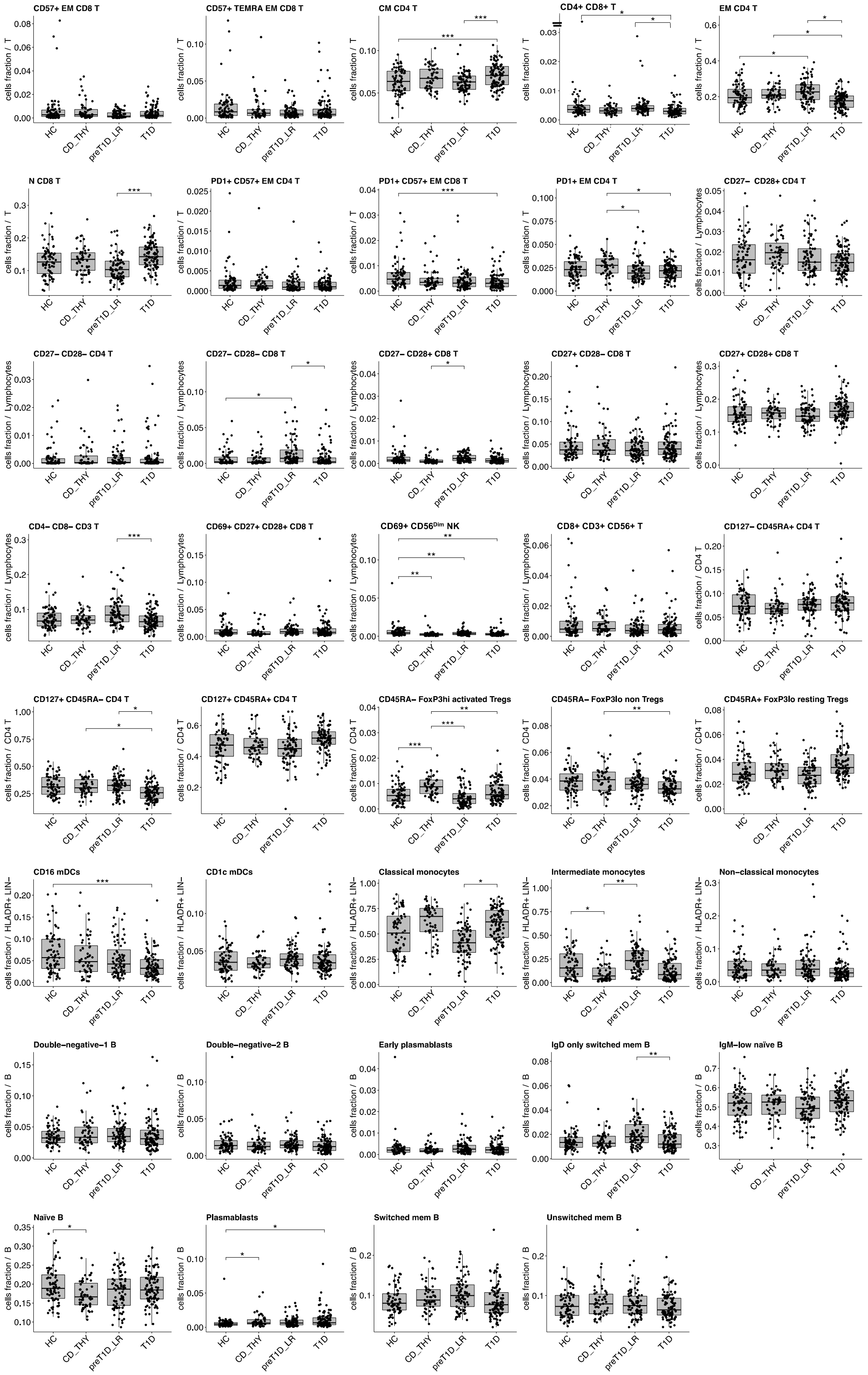

Supplementary Figure 7. Frequency of all the cell clusters identified with unsupervised clustering. Cell populations identified with the unsupervised clustering and not differentially represented in T1D compared to all other groups are shown. HC, healthy controls; CD_THY, celiac or thyroid diseases; preT1D_LR, relatives with 0-1 autoantibodies; T1D, type 1 diabetes. * $p < 0.05$, ** $p < 0.01$, *** $p < 0.001$.
